# Supplementary material for: Time-series transcriptome analysis identified differentially expressed genes in broiler chicken infected with mixed Eimeria species
Source: Front Genet. 2022 Aug 8;13:886781. doi: 10.3389/fgene.2022.886781 (PMC9393255; doi:10.3389/fgene.2022.886781)
Supplement: Supplementary file 2 [file DataSheet1.ZIP › 7dpi_KEGG.Gsea.1625115961950/KEGG_TYPE_II_DIABETES_MELLITUS.html]

Details for gene set KEGG\_TYPE\_II\_DIABETES\_MELLITUS[GSEA]

|  || Dataset | TMM\_7dpi\_gct\_format\_7dpi\_gct\_format.Class\_4dpi.cls #PC\_versus\_NC.Class\_4dpi.cls #PC\_versus\_NC\_repos |
| Phenotype | Class\_4dpi.cls#PC\_versus\_NC\_repos |
| Upregulated in class | 0 |
| GeneSet | KEGG\_TYPE\_II\_DIABETES\_MELLITUS |
| Enrichment Score (ES) | -0.4992175 |
| Normalized Enrichment Score (NES) | -1.5545756 |
| Nominal p-value | 0.014901048 |
| FDR q-value | 0.10292407 |
| FWER p-Value | 0.855 |
Table: GSEA Results Summary

  

Fig 1: Enrichment plot: KEGG\_TYPE\_II\_DIABETES\_MELLITUS      
 Profile of the Running ES Score & Positions of GeneSet Members on the Rank Ordered List

  

| SYMBOL | TITLE | RANK IN GENE LIST | RANK METRIC SCORE | RUNNING ES | CORE ENRICHMENT || 1 | GCK | na | 150 | 1.139 | 0.0349 | No |
| 2 | HK1 | na | 361 | 0.870 | 0.0536 | No |
| 3 | SOCS1 | na | 864 | 0.566 | 0.0352 | No |
| 4 | SOCS4 | na | 2946 | 0.133 | -0.1331 | No |
| 5 | CACNA1E | na | 3357 | 0.079 | -0.1640 | No |
| 6 | ADIPOQ | na | 3391 | 0.075 | -0.1636 | No |
| 7 | CACNA1G | na | 3825 | 0.024 | -0.1988 | No |
| 8 | PRKCD | na | 5230 | -0.124 | -0.3109 | No |
| 9 | CACNA1C | na | 5491 | -0.148 | -0.3264 | No |
| 10 | PRKCZ | na | 5653 | -0.165 | -0.3330 | No |
| 11 | HK3 | na | 6191 | -0.224 | -0.3685 | No |
| 12 | MAPK8 | na | 6464 | -0.250 | -0.3808 | No |
| 13 | IRS2 | na | 6867 | -0.293 | -0.4022 | No |
| 14 | IRS4 | na | 7368 | -0.348 | -0.4294 | No |
| 15 | MAPK9 | na | 7648 | -0.377 | -0.4370 | No |
| 16 | HK2 | na | 8169 | -0.442 | -0.4621 | No |
| 17 | IKBKB | na | 8465 | -0.479 | -0.4668 | No |
| 18 | PIK3CG | na | 8478 | -0.481 | -0.4478 | No |
| 19 | PRKCE | na | 8985 | -0.547 | -0.4672 | No |
| 20 | PKLR | na | 9010 | -0.550 | -0.4464 | No |
| 21 | SOCS2 | na | 9342 | -0.605 | -0.4488 | No |
| 22 | PIK3R2 | na | 9597 | -0.645 | -0.4432 | No |
| 23 | MTOR | na | 10269 | -0.763 | -0.4675 | Yes |
| 24 | MAPK3 | na | 10320 | -0.774 | -0.4394 | Yes |
| 25 | PIK3CB | na | 10335 | -0.777 | -0.4082 | Yes |
| 26 | PIK3CD | na | 10707 | -0.855 | -0.4036 | Yes |
| 27 | PIK3CA | na | 10920 | -0.912 | -0.3833 | Yes |
| 28 | PIK3R5 | na | 10941 | -0.918 | -0.3468 | Yes |
| 29 | CACNA1B | na | 10949 | -0.921 | -0.3091 | Yes |
| 30 | PIK3R3 | na | 11045 | -0.949 | -0.2775 | Yes |
| 31 | SOCS3 | na | 11181 | -0.994 | -0.2474 | Yes |
| 32 | PIK3R1 | na | 11265 | -1.023 | -0.2117 | Yes |
| 33 | ABCC8 | na | 11451 | -1.099 | -0.1814 | Yes |
| 34 | INSR | na | 11461 | -1.101 | -0.1364 | Yes |
| 35 | CACNA1D | na | 11943 | -1.649 | -0.1079 | Yes |
| 36 | MAPK10 | na | 12008 | -2.724 | 0.0002 | Yes |
Table: GSEA details [plain text format]

  

Fig 2: KEGG\_TYPE\_II\_DIABETES\_MELLITUS      
 Blue-Pink O' Gram in the Space of the Analyzed GeneSet

  

Fig 3: KEGG\_TYPE\_II\_DIABETES\_MELLITUS: Random ES distribution      
 Gene set null distribution of ES for **KEGG\_TYPE\_II\_DIABETES\_MELLITUS**
